# Schisandrin A in *Schisandra chinensis* Upregulates the LDL Receptor by Inhibiting PCSK9 Protein Stabilization in Steatotic Model

**DOI:** 10.4014/jmb.2306.06049

**Published:** 2023-10-31

**Authors:** Hyo-Jin Kim, Seon Kyeong Park, Soo Hyun Park, Yu Geon Lee, Jae-Ho Park, Jin-Taek Hwang, Min-Yu Chung

**Affiliations:** 1 Personalized Diet Research Group, Korea Food Research Institute, Wanju 55365, Republic of Korea; 2 Department of Food Biotechnology, University of Science & Technology, Daejeon 34113, Republic of Korea; 3 Department of Food and Nutrition, Gangseo University, Seoul 07661, Republic of Korea

**Keywords:** *Schisandra chinensis*, low-density lipoprotein receptor, proprotein convertase subtilisin/kexin 9, schisandrin A, HepG2 cells

## Abstract

*Schisandra chinensis* extract (SCE) protects against hypocholesterolemia by inhibiting proprotein convertase subtilisin/kexin 9 (PCSK9) protein stabilization. We hypothesized that the hypocholesterolemic activity of SCE can be attributable to upregulation of the PCSK9 inhibition-associated low-density lipoprotein receptor (LDLR). Male mice were fed a low-fat diet or a Western diet (WD) containing SCE at 1% for 12 weeks. WD increased final body weight and blood LDL cholesterol levels as well as alanine transaminase and aspartate aminotransferase expression. However, SCE supplementation significantly attenuated the increase in blood markers caused by WD. SCE also attenuated WD-mediated increases in hepatic LDLR protein expression in the obese mice. In addition, SCE increased LDLR protein expression and attenuated cellular PCSK9 levels in HepG2 cells supplemented with delipidated serum (DLPS). Non-toxic concentrations of schisandrin A (SA), one of the active components of SCE, significantly increased LDLR expression and tended to decrease PCSK9 protein levels in DLPS-treated HepG2 cells. High levels of SA-mediated PCSK9 attenuation was not attributable to reduced PCSK9 gene expression, but was associated with free PCSK9 protein degradation in this cell model. Our findings show that PCSK9 secretion can be significantly reduced by SA treatment, contributing to reductions in free cholesterol levels.

## Introduction

Cardiovascular disease (CVD) is one of the most significant causes of mortality and morbidity worldwide. Over the last ten years, the number of deaths due to CVD has increased by 12.5% globally [1, 2], with atherosclerosis being the leading cause of vascular disease and death. Atherosclerosis is caused by abnormal lipid metabolism, which leads to the accumulation of fatty streaks in the arterial layer, causing thickening and hardening of the arterial wall and resulting in narrowing of the vascular lumen [3]. Blood cholesterol, especially low-density lipoprotein (LDL), is a key driver of atherosclerosis [4]. Meta-analyses of large prospective observational studies have investigated the relationship between the risk of ischemic heart disease (IHD) and various lipid measures, including total cholesterol (TCHO), high-density lipoprotein (HDL) cholesterol, non-HDL cholesterol, triglycerides, and apolipoproteins [4-6]. These data suggest the existence of continuous positive log-linear relationships between normal blood levels of either total or LDL cholesterol (non-HDL cholesterol) and the risk of IHD. Meanwhile, a clear inverse correlation between HDL cholesterol and IHD risk has been found [5, 6].

Dietary intake of fat and cholesterol is an important environmental determinant of blood cholesterol levels, which can also be influenced by other factors, including exercise and genetics [7-10]. The consumption of dietary components that inhibit proprotein convertase subtilisin/kexin 9 (PCSK9) can positively influence lipid metabolism during the development and progression of atherosclerosis [11]. The role of PCSK9 in lipid metabolism has been extensively studied. PCSK9 binds to the LDL receptor, causing its degradation, thereby increasing circulating LDL levels in the blood. Cohort studies have demonstrated that circulating PCSK9 is a viable therapeutic target for reducing plasma LDL cholesterol levels to aid in the prevention of CVD [12, 13]. Observation of a large sample of healthy participants in the Brisighella Heart Cohort found that circulating PCSK9 levels were associated with increased arterial stiffness, and therefore potentially associated also with LDL cholesterol [13].

PCSK9 inhibitors bind to LDL receptors on the surface of liver cells, promoting receptor degradation and interfering with the clearance of LDL cholesterol from the bloodstream [14]. To date, specific natural substances that directly inhibit PCSK9 have not yet been reported. Widely known PCSK9 inhibitors are primarily synthetic compounds, such as evolocumab and alirocumab, but ongoing studies are exploring their safety [15]. The search for specific components of natural substances that directly inhibit PCSK9 remains an ongoing area of research and future findings may lead to new discoveries.

*Schisandra chinensis* is a perennial herb that is primarily distributed in northeastern China, Korea, eastern Russia, and Japan [16]. Traditional herbal medicines have long utilized various parts of *S. chinensis*, including its leaves and fruits, owing to their wide range of healing properties. Recently, there has been increasing interest in the potential pharmacological activities of *S. chinensis* fruit extract and its constituents, which include tannins, anthocyanins, and essential oils [17]. In particular, the major bioactive constituents of *S. chinensis* are dibenzocyclooctadiene lignans (gomisin A, gomisin B, gomisin C, gomisin N, schisandrin, deoxyschisandrin, and wuweizisu C) [17, 18]. In a previous study, dibenzocyclooctadiene lignans from *S. chinensis* were reported to have anti-inflammatory and antioxidant effects, and ameliorated cognitive function [17]. However, the regulatory effects of *S. chinensis* on LDL levels have not been fully elucidated. Therefore, we sought to determine whether the hypocholesterolemic activity of *S. chinensis* extract (SCE) is a result of LDL receptor (LDLR) upregulation and investigate the mechanism by which the major components of *S. chinensis* mediate LDLR-PCSK9 regulation in HepG2 cells and steatotic mice fed a Western diet (WD).

## Materials and Methods

Dulbecco’s modified Eagle’s medium (DMEM) and antibiotic antimycotic solution (100X) were purchased from Welgene Inc. (Republic of Korea). Delipidated serum (DLPS) was prepared from fetal bovine serum (FBS; Thermo Fisher Scientific, USA) as described previously [19, 20]. Isopropanol and chloroform were obtained from EMD Millipore (USA). Cycloheximide (CHX), bafilomycin A1 (BA1), 3-(4,5-dimethylthiazol-2-yl)-2,5-diphenyltetrazolium bromide (MTT), and MG132 were purchased from Sigma-Aldrich (USA). Dimethyl sulfoxide (DMSO) was purchased from Duchefa Biochemie (Netherlands).

### SCE Preparation

The fruit of *S. chinensis* was mixed with a 10-fold volume of 70% ethanol and agitated for 24 h at room temperature. The extract was then centrifuged at 8,000 × g for 30 min. The supernatant was lyophilized, stored at -20°C, and used in the experiment.

### In Vivo Model

The animal study was approved by the Institutional Animal Care and Use Committee at Wonkwang University (WKU16-21). Male C57BL/6 mice (7-week-old) were purchased from SAMTAKO Bio Korea (Republic of Korea) and acclimated for 1 week in an environmentally controlled facility. At 8 weeks of age, mice were randomized to control diet (CD; #98121701, Research Diets Inc., USA), WD (#D12079B, Research Diets INC), or WD formulated with 1% SCE (w/w; *n* = 10/group) for 12 weeks. Diets and water were provided ad libitum. Body weight and food intake were monitored and recorded once weekly. At termination, mice were sacrificed, and blood and liver samples were collected. Following washing with 0.9% saline solution, liver samples were stored at -80°C until analysis.

### Serum Analysis

Blood samples were stored at 4°C for 2 h and centrifuged before serum was collected. Serum lipids (triglyceride, total cholesterol and LDL cholesterol) and serum hepatic injury markers (alanine transaminase (ALT) and aspartate aminotransferase (AST)) were analyzed using enzymatic colorimetric assay kits (*Asan Pharmaceutical* Co., Ltd., Republic of Korea) with a Modular Analytics machine (Model PE, Roche Diagnostics, Germany).

### Cell Culture and Treatment

HepG2 cells (HB-8065) were purchased from the American Type Culture Collection (ATCC, USA), and maintained in DMEM containing 1% antibiotics and 10% (v/v) FBS at 37°C in a humidified atmosphere of 5% CO_2_ in an incubator. For sample (extract or chemicals) treatments, HepG2 cells were seeded (1.5 × 10^5^ cells/ml) in DMEM containing 1% antibiotics and 10% FBS (day 0). On the following day, cells were washed with DPBS twice, and the medium was changed to DMEM containing 1% antibiotics and 10% DLPS (day 1). After 24 h incubation, the medium was replaced with fresh DMEM containing 1% antibiotics and 10% DLPS supplemented with appropriate levels of SCE or schisandrin A (SA) up to 100 μM (day 2). Finally, 18 h later, cells were harvested for further analysis following washing with DPBS.

### Western Blotting

To evaluate hepatic protein expression, liver samples were homogenized with radioimmunoprecipitation assay (RIPA) buffer (Elpis Biotech, Inc., Republic of Korea) containing protease and phosphatase inhibitor (Roche), before being placed on ice for 30 min with intermittent vortexing. Following centrifugation (20,000 ×*g*, 30 min, 4°C), the cell lysates were collected. To evaluate cellular protein expression, HepG2 cells were cultured as described in the previous section. Following treatment with SCE (up to 200 μg/ml) or SA (up to 80 μM) as described previously, the cells were washed with DPBS and lysed with RIPA buffer, and placed on ice for 30 min. Following centrifugation (15,000 ×*g*, 30 min, 4°C), the supernatant was collected.

Collected whole cell lysates were then used to analyze total protein content using a BCA kit (Pierce, USA). After quantification, 20 μg of protein was separated by 10% SDS-PAGE. Proteins were transferred to a nitrocellulose membrane (GE Healthcare BioScience, USA). The membrane was blocked in PBS containing 0.05% Tween 20 and 5% non-fat dried milk for 1 h at room temperature, and then incubated in blocking solution with primary antibodies against PCSK9 (Circulex, Medical & Biological Laboratories Co., Ltd., USA), LDLR (BioVision Inc., Milpitas, CA, USA), SREBP2 (Cayman Chemical, USA), hepatic nuclear factor (HNF)-1α (Cell Signaling Technology, USA) and β-actin (Bethyl Laboratories, USA) for 1 h at room temperature. After washing with PBS containing 0.05% Tween 20 and 5% non-fat dried milk, the membrane was incubated in horseradish peroxidase secondary antibody (Thermo Scientific, USA). Target proteins were visualized with a FUSION SOLO 6S imaging system (Vilber Lourmat, AZC de Lamirault, France) with an enhanced chemiluminescence detection reagent (Thermo Scientific). Target protein expression was normalized to β-actin.

### Cell Viability

To examine viability of the HepG2 cells, a colorimetric assay using MTT was performed. To determine non-toxic concentrations for the cells, HepG2 cells were cultured with SCE (200, 400 μg/ml) or SA (10‒100 μM) in DMEM containing 10% DLPS. After an additional 18 h incubation, MTT solution (final concentration: 0.5 mg/mL) was added in each well. After a further 2 h incubation, a mixture of isopropanol and DMSO (1:1) was added to the wells before incubation for 5 min with gentle shaking. Sample absorbance was measured at 570 nm using a microplate reader (SpectraMax M2 microplate reader; Molecular Devices, USA).

### Enzyme-Linked Immunosorbent Assay (ELISA)

HepG2 cells were cultured as described in Cell Culture and Treatment. Cells were treated with SCE or SA, and the plate was incubated for another 18 h. After incubation, both cell pellets and media were collected. Pellets were rinsed with DPBS and lysed with RIPA buffer (Elpis Biotech, Inc., Republic of Korea) containing protease and phosphatase inhibitor (Roche, Switzerland) in ice, before vortexing, centrifuging (15,000 ×*g*, 30 min, 4°C), and collection of the supernatant as a whole cell lysate. ELISA kits were purchased from Cell Biolabs Inc., (USA) and analysis of PCSK9 (STA 385) and LDLR (STA 386) was performed per the manufacturer’s instructions. Quantification of the protein in samples was determined in triplicate by BCA protein analysis (Pierce BCA Protein Assay Kit, Thermo Scientific, USA). Protein analysis was also performed according to the manufacturer’s instructions. PCSK9 and LDLR levels were normalized to total protein content.

### LDL Uptake Assay

To examine LDL uptake at the cellular level, HepG2 cells were seeded (3 × 10^4^ cells/well) in 96-well plates and treated as previously described. We used an LDL uptake kit (Cayman Chemical) and performed the assay according to the manufacturer’s instructions. At the end of the treatment period, we added a fluorescent probe, DyLight 550, and incubated it for 12 h at 37°C in a humidified atmosphere of 5% CO_2_. Cells were rinsed and fixed for immunofluorescence staining. After washing with buffer, images were captured using a fluorescence microscope (IX71; Olympus, Japan).

### Quantitative Real-Time Polymerase Chain Reaction (qRT-PCR)

HepG2 cells were maintained, seeded in 6-well plates, and cultured as described. On day 2, the cells were incubated with DMEM containing 1% antibiotics and 10% DLPS supplemented with SA (20, 40, and 80 μM), and incubated for an additional 18 h. Cells were washed with DPBS two times, and pellets were collected following centrifugation. Total RNA was extracted from pellets using TRIzol (Invitrogen, USA). cDNA was synthesized using a reverse transcription kit (Bio-Rad) and gene expression was quantified with SYBR Green PCR master mix (Thermo Fisher Scientific) on a Bio-Rad CFX384. Primers used for qRT-PCR are provided in Table 1. Target gene expression was quantified relative to GAPDH, using the 2^-ΔΔCT^ method. All reactions were carried out in triplicate.

### Protein Stability

To evaluate PCSK9 protein stability regulated by SA, cells were treated simultaneously with SA and cycloheximide (CHX). In brief, HepG2 cells were cultured and seeded as described previously, followed by incubation in fresh DMEM supplemented with DLPS for 24 h (day 1). On the following day (day 2), cells were treated with SA at 0 or 80 μM. On day 3, cells were treated with protein synthesis inhibitor CHX (C4895, Sigma-Aldrich) at 5 μg/ml. Cells were then harvested at 0, 1, 2, 4, and 8 h post incubation with CHX. Western blotting was performed as described above using all the collected pellets followed by protein lysis.

To evaluate the effect of SA against lysosomal and proteasomal degradation inhibition, HepG2 cells were cultured and seeded (day 0) as described previously. On the following day (day 1), the cells were incubated with DMEM supplemented with 10% DLPS, followed by treatment (day 2) with chemicals [bafilomycin A1 (BA1; Sigma-Aldrich) at 0 or 50 nM or MG132 (Sigma-Aldrich) at 0 or 1 μM] and SA at 0 or 80 μM. Following 24 h incubation on day 3, the cells were washed with DPBS, harvested, and centrifuged, with the upper layer removed to collect whole cell lysate.

### Filipin Staining Assay

To measure for cholesterol, HepG2 cells were cultured, seeded, and treated as described in Cell Culture and Treatment section. After 18 h of further incubation at day 2, cells were washed with DPBS 3 times, and fixed with 3.7% paraformaldehyde for 1 h. Following washing with DPBS 3 times, glycine solution dissolved in DPBS (100 μl) was added to each well to remove residual paraformaldehyde. Filipin solution (50 μg/ml) was then added to each well and incubated for 2 h at room temperature, followed by rinsing with DPBS 3 times. Fluorescence intensity was measured at 360/480 nm (excitation/emission) using a SpectraMax M2 microplate reader (Molecular Devices).

### Statistical Analysis

Data (means ± standard error of mean (SEM)) were analyzed using GraphPad Prism software (version 7.0; USA). Student’s *t*-test was used to evaluate differences in markers between CD and WD, and WD and WD+CBE, following 12 weeks’ feeding. All analyses were considered statistically significant at *p* < 0.05.

## Results

### SCE Supplementation Attenuates WD-Induced Increases in Body Weight, Serum Lipid Profiles, and Hepatic Injury Markers, as well as LDLR Protein Expression

**Fig. 1:**
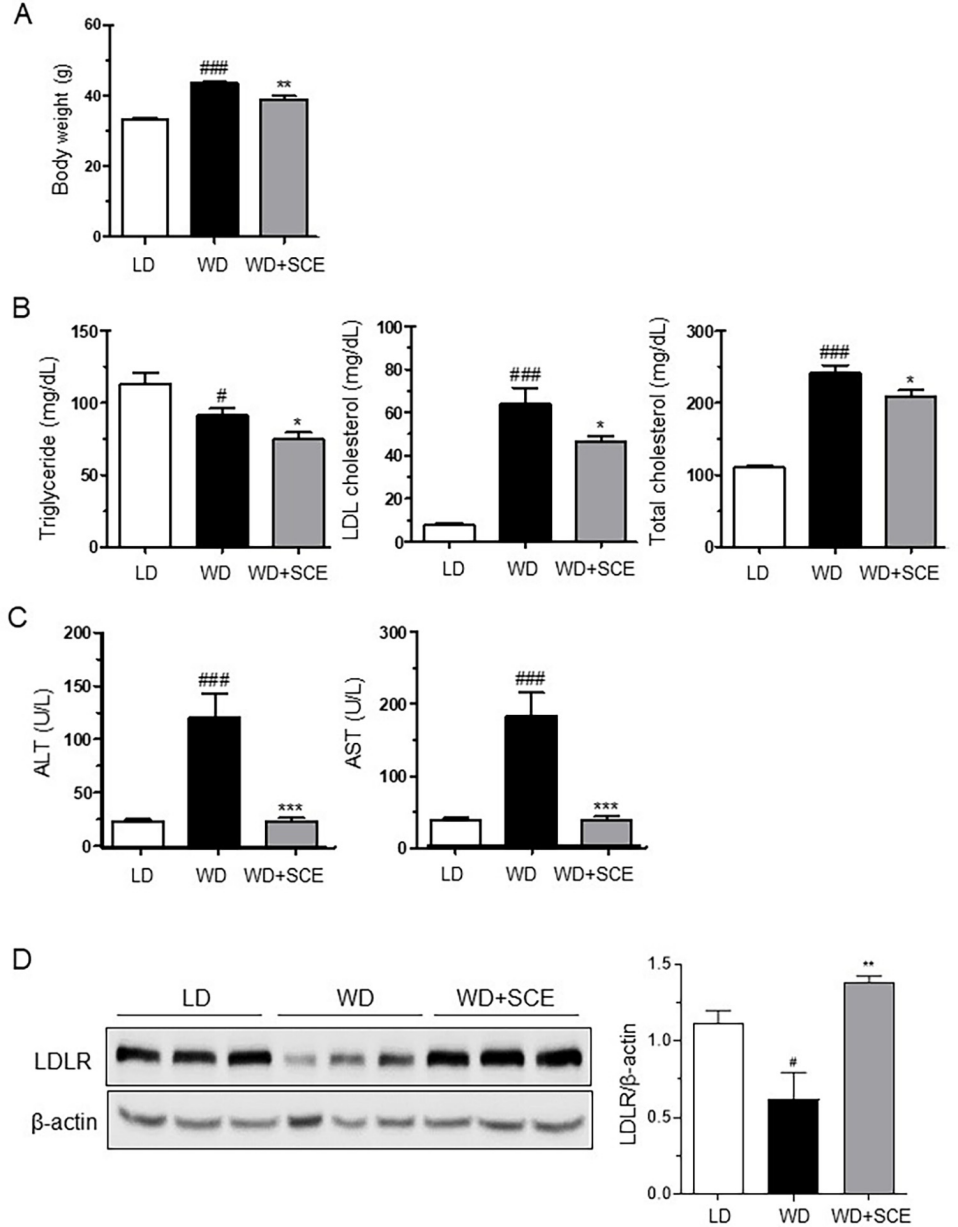
*Schisandra chinensis* extract (SCE) attenuates final body weight, serum lipid profiles, hepatic injury markers, and low-density lipoprotein receptor (LDLR) expression in liver tissues of Western diet (WD)-fed obese mice. Changes in body weight (**A**) serum lipid profiles (TG, TCHO, and LDL-C) (**B**) serum hepatic injury markers (ALT and AST) (**C**) and LDLR protein expression (**D**) in the liver tissue of WD-fed obese mice. The results are expressed as the mean ± SEM. # indicates a significant difference compared to the low-fat diet (LD) group (#*p* < 0.05, ##*p* < 0.01, ###*p* < 0.001). * indicates a significant difference compared to the WD group (*p < 0.05, **p < 0.01, ***p < 0.001). LD, low-fat diet; WD+SCE, WD plus *Schisandra chinensis* extract, TG; triglyceride, TCHO; total cholesterol, LDL-C; LDL-cholesterol, ALT; alanine transaminase, AST; aspartate aminotransferase.

SCE (1%, w/w) supplementation significantly attenuated the final body weight of WD-fed obese mice compared with that of the controls (Fig. 1A). Twelve weeks of SCE supplementation also significantly reduced total cholesterol, LDL cholesterol, and TG levels in the serum compared with WD feeding in obese mice (Fig. 1B). To examine the effect of WD and low-fat diet (LD) feeding for 12 weeks on liver injury in mice, serum ALT and AST activities were measured. We observed that the WD significantly increased both ALT and AST activity, which were significantly decreased by SCE supplementation (Fig. 1C). ALT and AST activities in the serum were increased approximately 5- and 2-fold by WD feeding, respectively, and were almost normalized in the LD group with SCE supplementation.

LDLR is a key regulator of cholesterol metabolism. LDLR knockout (Ldlr-/-) Leiden mice are widely used to study dyslipidemia, obesity, and nonalcoholic steatohepatitis following high-fat diet feeding [21, 22]. Therefore, we examined whether the SCE-mediated reduction in LDL cholesterol levels were attributable to LDLR upregulation in the livers of these obese mice (Fig. 1D). We found that WD feeding markedly attenuated LDLR protein expression, which was reversed by SCE supplementation. LDLR protein expression in the WD+SCE group was similar to that observed in LD controls. Therefore, we deduced that SCE increased LDLR protein expression, contributing to reduced serum LDL cholesterol levels in obese mice.

### SCE Increases LDLR Protein Expression and Reduces PCSK9 Secretion in DLPS-Treated HepG2 Cells

**Fig. 2:**
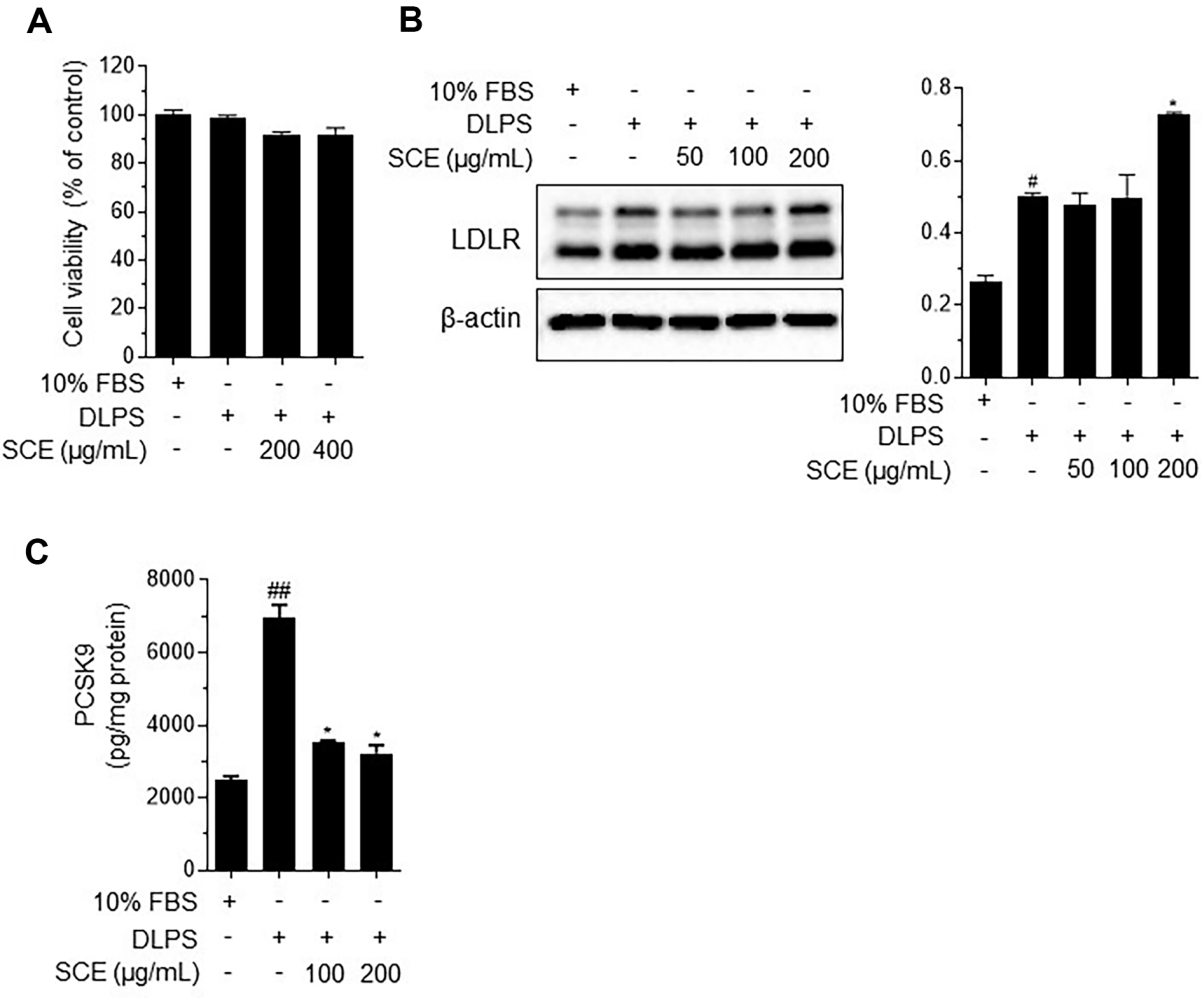
*Schisandra chinensis* extract increases low-density lipoprotein receptor (LDLR) expression and inhibits proprotein convertase subtilisin/kexin 9 (PCSK9) secretion in delipidated serum (DLPS)-induced lipid-deprived conditions. Cell viability (**A**) LDLR protein expression in HepG2 cells (**B**) and PCSK9 levels in the media (**C**). The results are expressed as the mean ± SEM. # indicates a significant difference compared to the normal control (#*p* < 0.05, ##*p* < 0.01, ###*p* < 0.001). * indicates a significant difference compared to DLPS-treated HepG2 cells (*p < 0.05, **p < 0.01, ***p < 0.001). FBS, fetal bovine serum; SCE, *Schisandra chinensis* extract.

The regulation of hypercholesterolemia, attributed to LDLR upregulation by SCE treatment, was further examined in HepG2 cells. DLPS was used to simulate lipid-deprived conditions for the purpose of PCSK9 upregulation [19, 20]. SCE, at concentrations up to 400 μg/ml, did not affect the viability of DLPS-treated HepG2 cells (Fig. 2A). We then measured LDLR protein expression in DLPS-treated cells and found that SCE at a concentration of 200 ug/ml, markedly increased LDLR expression compared to the DLPS-treated cells (Fig. 2B). Next, we measured PCSK9 secretion using ELISA. DLPS significantly increased PCSK9 secretion, which was significantly reduced by SCE at concentrations of 100 and 200 μg/ml (Fig. 2C). These findings suggest that SCE inhibits PCSK9 secretion, thereby preventing LDLR degradation and likely contributing to the reduction of free cholesterol levels in HepG2 cells.

### Schisandrin A Induces LDLR Upregulation and PCSK9 Inhibition in DLPS-Treated HepG2 Cells

**Fig. 3:**
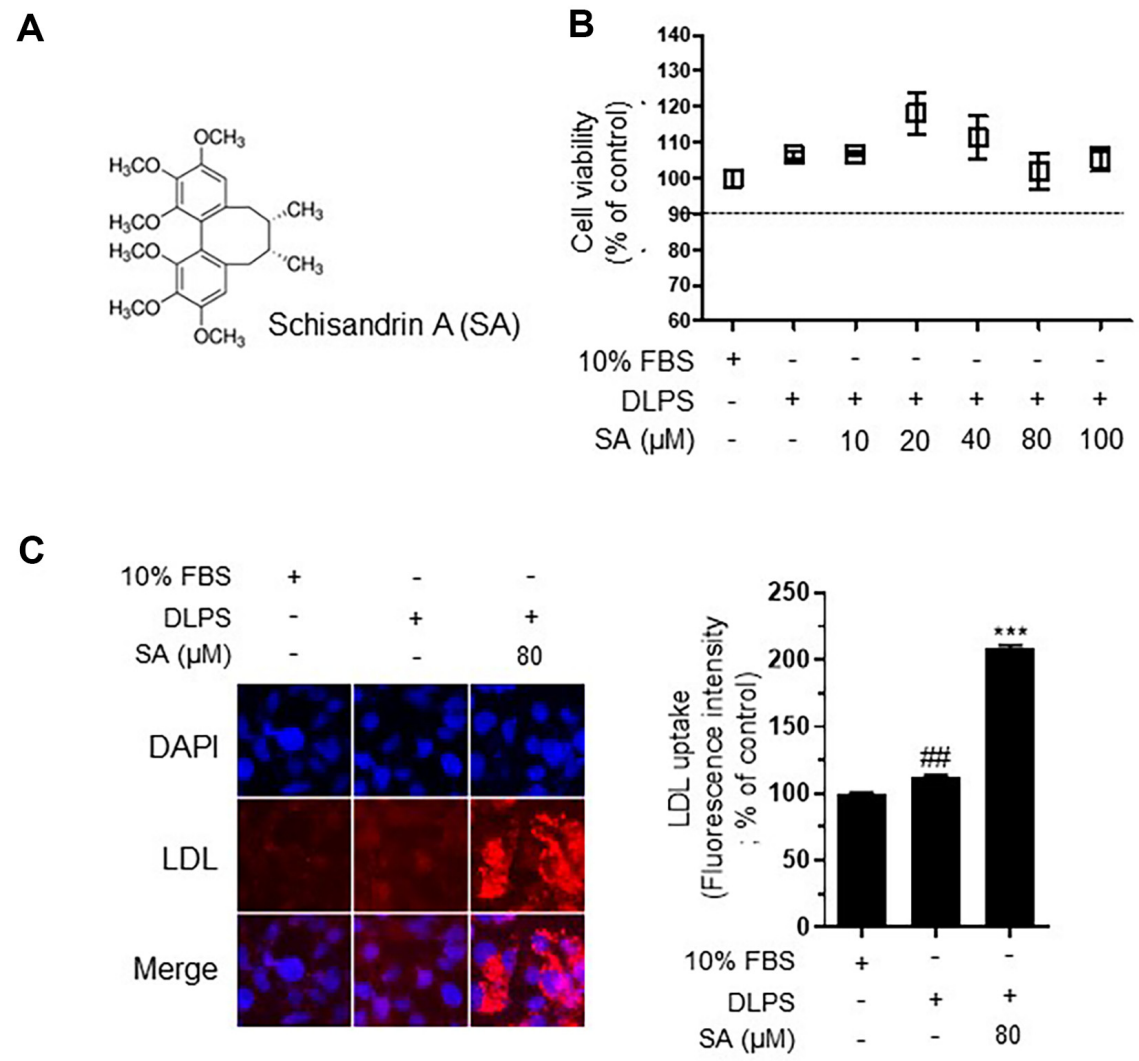
Schisandrin A (SA) increases low-density lipoprotein (LDL) uptake in delipidated serum (DLPS)- treated HepG2 cells. Chemical structure of SA (**A**) cell viability (**B**) and representative immunofluorescence images and fluorescence intensity of the LDL-cholesterol uptake assay (**C**) in HepG2 cells. The results are expressed as the mean ± SEM. # indicates a significant difference compared to the normal control (#*p* < 0.05, ##*p* < 0.01, ###*p* < 0.001). * indicates a significant difference compared to DLPS-treated HepG2 cells (*p < 0.05, **p < 0.01, ***p < 0.001).

We examined the components of SCE to determine which of them contribute to the observed hypocholesterolemic activity of PCSK9 inhibition. SA is a major bioactive component of *S. chinensis* (Fig. 3A) [16]. We measured cell viability using the MTT assay and found that SA, at a concentration up to 100 μM, had no observable cytotoxic effects in the DLPS-treated HepG2 cells (Fig. 3B). SA (80 μM) significantly increased LDL uptake in DLPS-treated HepG2 cells (Fig. 3C). These findings suggest that the hypocholesterolemic effect of SCE is likely attributable to the regulation of the LDLR-PCSK9 protein by SA.

### SA Significantly Regulates LDLR/PCSK9 Protein Expression but Not PCSK9 Gene Expression

**Fig. 4:**
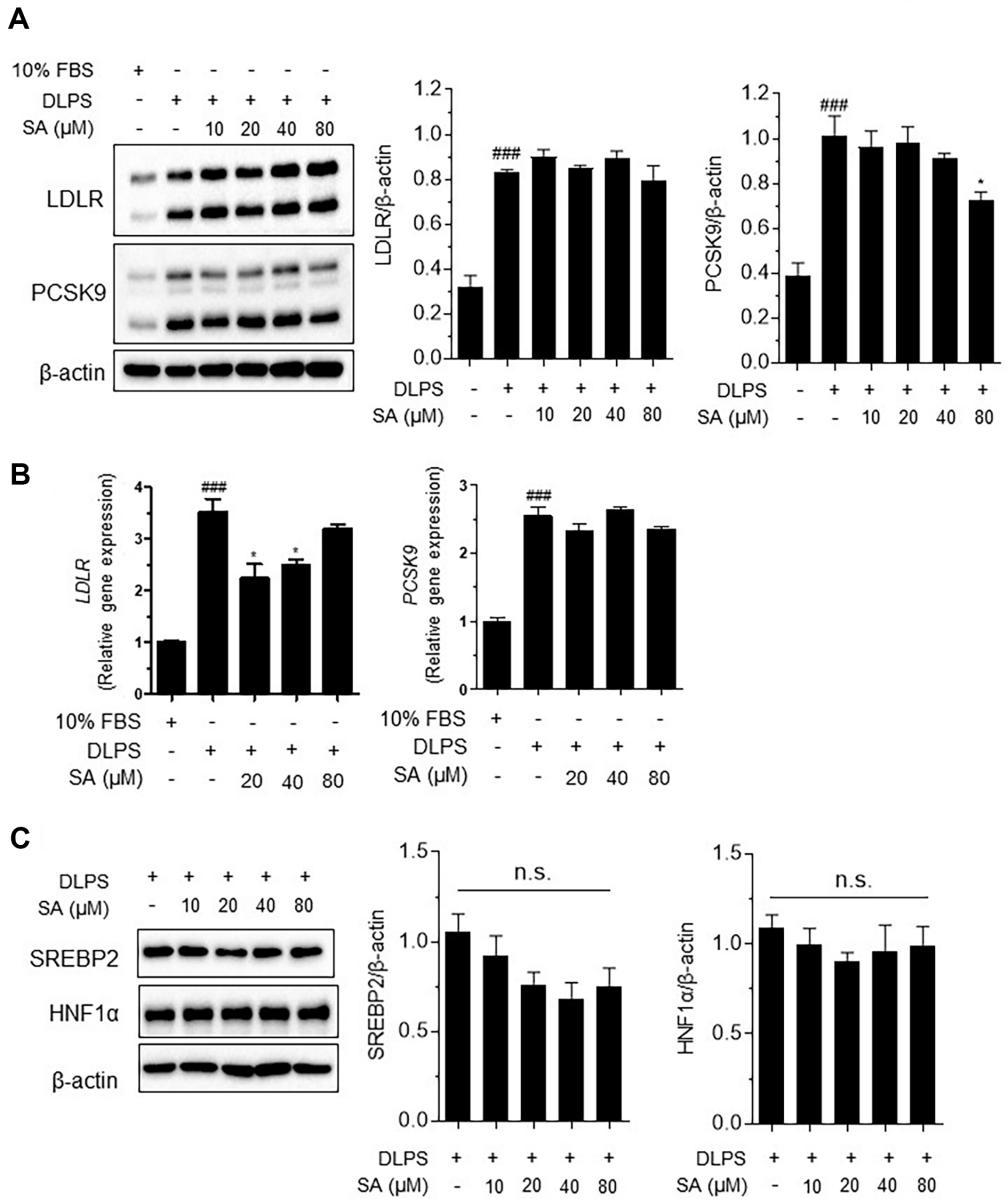
Schisandrin A (SA) regulates low-density lipoprotein receptor (LDLR)- proprotein convertase subtilisin/kexin 9 (PCSK9) protein expression but is not associated with their gene expression or the upregulation of hepatic nuclear factor (HNF)-1α and SREBP2 protein expression in delipidated serum (DLPS)-treated HepG2 cells. LDLR and PCSK9 protein expression (**A**) LDLR and PCSK9 gene expression (**B**) and SREBP2 and HNF-1α protein expression (**C**) in HepG2 cells. The results are expressed as the mean ± SEM. # indicates a significant difference compared to the normal control (#*p* < 0.05, ##*p* < 0.01, ###*p* < 0.001). * indicates a significant difference compared to DLPS-treated HepG2 cells (*p < 0.05, **p < 0.01, ***p < 0.001). n.s., no significant difference.

LDLR and PCSK9 protein expression levels were confirmed using western blot analysis. SA (10, 20, 40, and 80 μM) increased LDLR protein expression, and 80 μM SA inhibited PCSK9 expression in DLPS-treated HepG2 cells (Fig. 4A).

In HepG2 cells, LDLR gene expression was significantly increased by DLPS treatment, which was in turn significantly increased by treatment with SA (80 μM) (Fig. 4B). DLPS-mediated increases in PCSK9 gene expression were not affected by any level of SA treatment (Fig. 4B).

### LDLR-PCSK9 Protein Modulation by SA Is Not associated with HNF-1α or SREBP2 Regulation in HepG2 Cells

The expression levels of SREBP2 and HNF-1α were examined by western blotting. We found that DLPS treatment downregulated SREBP2 protein expression, while SA slightly downregulated SREBP2 (Fig. 4C). Hepatic nuclear factor HNF-1α is a transcription factor that regulates PCSK9 independently of LDLR [23]. We found that HNF-1α protein expression was unaffected by DLPS or SA treatments (Fig. 4C). These findings suggested that the hypocholesterolemic effect of SA was not associated with the regulation of transcription factors that mediate LDLR and PCSK9 protein expression.

### SA Induces PCSK9 Destabilization, thereby Preventing LDLR Lysosomal Degradation in HepG2 Cells

**Fig. 5:**
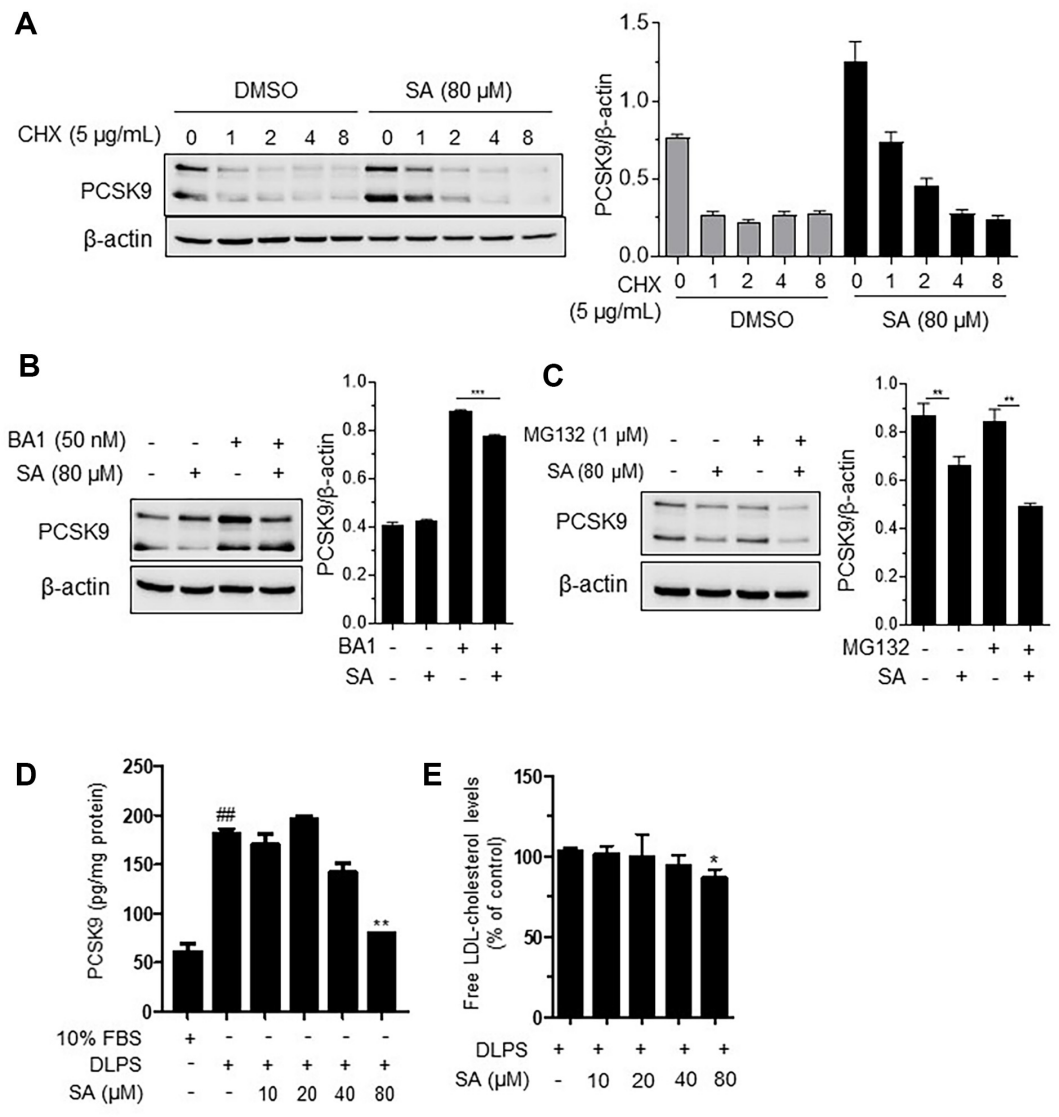
Schisandrin A (SA) effectively blocks proprotein convertase subtilisin/kexin 9 (PCSK9) secretion in delipidated serum (DLPS)-treated HepG2 cells. PCSK9 protein expression in the presence or absence of cycloheximide (CHX) (**A**) bafilomycin A1 (BA1) (**B**) and MG132 (**C**) in HepG2 cells. Released PCSK9 levels into the medium (**D**) and free low-density lipoprotein-cholesterol levels in the medium (**E**). The results are expressed as the mean ± SEM. # indicates a significant difference compared to the normal control (#*p* < 0.05, ##*p* < 0.01, ###*p* < 0.001). * indicates a significant difference compared to DLPS-treated HepG2 cells (*p < 0.05, **p < 0.01, ***p < 0.001).

To examine PCSK9 protein stabilization by SA treatment, cells were simultaneously treated with CHX, an inhibitor of new protein synthesis, and SA. When PCSK9 translation was blocked, PCSK9 protein expression reduced in a time-dependent manner up to 8 h, which was also consistently observed with 80 μM of SA treatment (Fig. 5A), suggesting that SA does not contribute to stabilization of the PCSK9 protein.

Circulating PCSK9 binds to LDLR and is transported to lysosomes for degradation [24]. As expected, treatment with the lysosomal degradation inhibitor, BA1, increased PCSK9 protein expression in DLPS-treated cells. However, SA enhanced the BA1-mediated increase in PCSK9 protein expression in DLPS-treated HepG2 cells (Fig. 5B). These results indicated that SA contributes to the stabilization of PCSK9 protein binding to LDLR in lysosomes.

PCSK9 stabilization by SA was further evaluated using the proteasome degradation inhibitor MG132 in DLPS-treated HepG2 cells (Fig. 5C). SA co-treated with MG132 markedly decreased PCSK9 protein levels, suggesting that SA destabilizes free PCSK9 in HepG2 cells treated with DLPS.

Next, we sought to confirm whether SA caused the destabilization of free PCSK9, thereby blocking PCSK9 secretion. Levels of PCSK9 secreted into the medium were measured using ELISA. DLPS was found to significantly increase PCSK9 secretion, which was in turn significantly reduced by SA (80 μM) treatment in HepG2 cells (Fig. 5D). SA also reduced free LDL-cholesterol (LDL-C) levels in DLPS-treated HepG2 cells (Fig. 5E). Taken together, these observations support the previous finding that SA destabilizes free PCSK9, leading to the attenuation of circulating PCSK9, thereby protecting LDLR from protein degradation and subsequently reducing cholesterol levels in HepG2 cells.

## Discussion

Cholesterol homeostasis is an essential, yet complex, aspect of metabolic regulation [25, 26]. Dietary cholesterol is absorbed into the intestine, whereas endogenous cholesterol is synthesized in a number of tissues, primarily in the liver. Lipoprotein particles play an important role in maintaining cholesterol homeostasis in the liver. Lipoproteins belong to several categories, including chylomicrons, very low-density lipoproteins (VLDL), LDL, and HDL.

Chylomicrons transport esterified dietary cholesterol from the intestine to the liver. VLDL is produced in the liver by combining triglycerides (TG) and cholesterol ester (CE) with apolipoproteins and other lipoproteins, and secreted into the bloodstream to be transported to peripheral tissues, such as muscle and adipose tissues. During this process, TG was removed from VLDL, converting to LDL containing more CE than TG [25, 27]. LDL is taken up by tissue expressing the apo B-containing receptor, and the CE transported by LDL becomes available for use by cells. Excess LDL (or oxidized LDL) often accumulates in cells via macrophages to form foam cells, which are a primary component of plaques during the development of atherosclerosis. Oxidized LDL is taken up by lesional macrophages before delivery to lysosomes, where oxidized LDL is hydrolyzed to free cholesterol by lysosomal acid lipase [28]. Free cholesterol can cause inflammation, is associated with various chronic diseases, and is transported from the lysosome to the endoplasmic reticulum, where it is esterified and stored as lipid droplets under normal conditions, whereas both CE and free cholesterol accumulate in cells where cholesterol trafficking occurs [29, 30]. Accumulated CE (as lipid droplets) causes the foamy morphology of macrophages, contributing to the production of atherosclerotic plaques. Together with the buildup of free cholesterol, which causes inflammation, these factors increase the risk of atherosclerosis [31].

Several strategies mediated by food compounds or diet plans can reduce circulating LDL cholesterol levels. In addition to enhancing LDLR expression, cholesterol excretion or absorption can be mediated when LDL cholesterol reaches critical levels. A meta-analysis of 14 randomized controlled trials with 1,136 individuals indicated that the consumption of green tea and its extracts significantly reduced fasting serum total cholesterol and LDL cholesterol levels (but not that of HDL cholesterol) in adults [32]. There are several intermediates through which flavonoids attenuate LDL cholesterol levels, which are often LDLR/PCSK9-independent. The underlying mechanism of PCSK9 inhibition may involve the upregulation of LDLR recycling and LDL uptake, providing a scientific basis for the prevention and treatment of steatosis. Therefore, our study focused on evaluating the potential of SCE and its constituents, which are natural substances, to regulate LDL levels via the LDLR-PCSK9 pathway.

*S. chinensis* has traditionally been used as a medicinal herb and was recently reported to alleviate cardiovascular symptoms associated with menopause [33]. Its possible mechanisms are reported to regulate NO signaling and vascular smooth muscle cells through dephosphorylation of the myosin light chain; however, specific studies related to cholesterol regulation have not been reported. According to our results (Fig. 1), SCE effectively decreased body weight in WD-fed obese mice and attenuated total and LDL cholesterol levels in the serum, which was associated with SCE-mediated increases in LDLR protein in liver tissue. In addition, SCE increased LDLR protein expression via PCSK9 inhibition in DLPS-treated HepG2 cells (Fig. 2). LDL is a combination of proteins and lipids that transport cholesterol in the bloodstream. LDL is synthesized in the liver and transported via circulation to other tissues and cells [34]. However, elevated LDL levels can increase the risk of atherosclerotic CVDs. LDLR, a protein present in the liver and other tissues, plays a crucial role in recognizing LDL in the bloodstream and facilitating the transport of cholesterol into cells. The liver regulates LDLR expression to control cholesterol metabolism [35]. When LDL levels are elevated, liver cells increase the expression of LDLR to enhance the uptake and clearance of cholesterol. Furthermore, PCSK9 plays an important role in LDLR regulation. PCSK9 is synthesized in the liver and secreted into the bloodstream. Circulating PCSK9 binds to the LDLR complex and induces intracellular degradation, leading to reduced LDL removal. Consequently, this leads to a reduced clearance of cholesterol through LDLR in the liver, resulting in elevated levels of LDL in the bloodstream [36]. Therefore, blocking PCSK9 with specific inhibitors has been proposed as a potential lipid-lowering strategy. Recently, PCSK9 inhibitors have been developed to lower LDL levels in the bloodstream. These medications reduce the risk of CVDs associated with high LDL levels. Alirocumab and evolocumab are FDA/EMA-approved monoclonal antibodies for PCSK9 inhibition; however, they have some disadvantages, such as high cost, subcutaneous administration, potential immunogenicity during long-term treatment, and an undefined safety profile [37].

Several food extracts with PCSK9 inhibitory activity have been identified. For instance, extracts of *Allium fistulosum* [38], *Allium tuberosum* [39], and *Capsella bursa-pastoris* [20] have been shown to inhibit PCSK9 levels in HepG2 cells cultured under lipid-deprived conditions and in steatotic WD-fed mice. Evidence shows that a number of flavonoids can also act as PCSK9 inhibitors. For example, naringin downregulates PCSK9/IDOL, facilitating reverse cholesterol transport in ApoE-/- mice fed a high-fat diet [40]. Similarly, butein [41] and epigallocatechin gallate [42] inhibit PCSK9 and upregulate LDLR by regulating HNF-1α in HepG2 cells and rats fed a high-fat diet, respectively. In addition, quercetin protects against atherosclerosis via regulation of PCSK9 as well as LXRα and ABCA1 [43]. Silibinin A also significantly reduces PCSK9 gene and protein expression in statin-treated HepG2 cells [44], and pinostrobin inhibits PCSK9 expression in HepG2 cells [45]. Based on these reports, natural materials from food extracts have demonstrated their potential use as PCSK9 inhibitors. *S. chinensis* is a rich source of phytochemicals, such as phenolic compounds (*e.g.*, anthocyanins and flavonols) and phytosterols (*e.g.*, lignans), which have strong antioxidative and anti-inflammatory effects in in vitro and in vivo models. In particular, lignans are used by traditional Oriental clinicians to treat several diseases, including hepatitis and cancer. Here, we confirmed the LDL-regulatory effect of SCE via PCSK9 inhibition and focused on the mechanism of SA as a major bioactive component of SCE. As shown in Fig. 3, SA reduced PCSK9 levels and significantly increased LDL uptake in DPLS-treated HepG2 cells, suggesting that SA is involved in LDL regulation. Lignans are composed of phenylpropane dimers containing two propane residues, C6–C3, and are found in various plant parts [46]. *S. chinensis* contains lignans such as angeoyl-/tigloylgomisin, gomisin, and schisandrin isomers as major bioactive compounds. In particular, lignans from *Schisandra* spp. are called schisandrins [46]. Kim *et al*. [47] analyzed the content of lignans (schisandrin, 25.95 mg/g; gomisin A, 2.51 mg/g; and gomisin M2, 2.17 mg/g) from the ethanol extract of *S. chinensis*, and reported that schisandrin was the most prevalent. SA has been reported to have anti-oxidant, anti-inflammatory, anti-cancer and hepatoprotective effects [48-50]. In particular, SA intake attenuated plasma free fatty acid and TG level through the regulation of lipid metabolism and oxidative stress on high fat/high cholesterol-induced nonalcoholic fatty liver disease mice [51]. On the other hand, research on the mechanism of LDL-C regulation is unclear. Our findings shed light on the mechanisms through which SCE and its active compound SA regulate LDL-C attenuation. SA increases LDLR protein expression and attenuates PCSK9 protein expression.

In our study, SA reduced PCSK9 stabilization in the proteasome, a likely mechanism by which it contributes to the protection of LDLR against protein degradation by lysosomes (Fig. 5). Destabilization of PCSK9 aims to increase the availability of functional LDLRs and promote the clearance of LDL-C from the bloodstream. This can be beneficial for the management of high LDL-C levels, reducing the risk of CVDs and improving lipid profiles.

Taken together, these observations suggest that SA effectively blocks the free (not bound to LDLR) PCSK9 form, thereby recycling it back to the cell membrane of LDLR. Sun *et al*. [52] demonstrated the anti-hyperlipidemic effects of SCE through the analysis of serum biochemistry (triglycerides, LDL-C, total cholesterol, and HDL-C) and metabolomics, including the tricarboxylic acid cycle, ketone bodies, cholesterol, and fatty acid metabolism. The underlying mechanism may involve the downregulation of LXRα/SREBP-1c/FAS/ACC and SREBP2/HMGCR signaling pathways in liver tissue. In contrast to these reports, our findings demonstrate that SA inhibits PCSK9 secretion and reduces free cholesterol levels but is not associated with HNF-1α or SREBP2. In addition, some phytochemicals are confirmed to inhibit PCSK9 via different mechanisms [41, 42]. For example, epigallocatechin gallate inhibited PCSK9 secretion, and quercetin and pinostrobin regulated PCSK9 expression by inhibiting the autocatalytic processing and maturation of PCSK9 in the endoplasmic reticulum [42]. However, the PCSK9 regulatory effect and mechanism of action of natural compounds remain unclear. Therefore, further studies are warranted to investigate the clinical relevance of natural compound-mediated LDL cholesterol-lowering, and the hypocholesterolemic activity in particular requires further investigation in animal model or patients receiving statin medication.

Conclusively, SA, a natural bioactive compound found in *S. chinensis*, contributes to the destabilization of free PCSK9 protein, thereby protecting LDLR from lysosomal degradation. Together, these effects contributed to the inhibition of PCSK9 secretion in HepG2 cells. Our findings offer an opportunity for the development of novel therapeutic agents using *S. chinensis* and SA, which can be used to treat CVDs by inhibiting PCSK9 secretion.

## Figures and Tables

**Table 1:** Sequence list of qRT-PCR primers.

Primer name	Direction	Sequence (5'->3')
PCSK9	Forward	GGCAGGTTGGCAGCTGTTT
	Reverse	CGTGTAGGCCCCGAGTGT
LDLR	Forward	AGGAGACGTGCTTGTCTGTC
	Reverse	CTGAGCCGTTGTCGCAGT
